# Gefitinib-based therapy in refractory hepatocellular carcinoma with EGFR overexpression: a spatial profiling-guided case report

**DOI:** 10.3389/fimmu.2026.1891806

**Published:** 2026-09-24

**Authors:** Qin Xu, Xiaofen Li, Rui Zhu, Zirui Wang, Nuerye Tuerhonga, Chengjian Zhao, Yu Yang

**Affiliations:** 1 Department of Medical Oncology, Cancer Center, West China Hospital, Sichuan University, Chengdu, Sichuan, China; 2 Division of Abdominal Tumor Multimodality Treatment, Cancer Center, West China Hospital, Sichuan University, Chengdu, Sichuan, China; 3 State Key Laboratory of Biotherapy and Cancer Center, Sichuan University and Collaborative Innovation Center, West China Hospital, Chengdu, Sichuan, China

**Keywords:** cutaneous metastasis, EGFR, hepatocellular carcinoma, resistance, tumor microenvironment

## Abstract

Current systemic treatments for advanced hepatocellular carcinoma (HCC) such as antiangiogenic targeted therapies, immune checkpoint inhibitors (ICIs), and their combinations have shown benefit, but widespread drug resistance severely limits patient outcomes and underscores an urgent need for effective post-progression strategies. We herein present a case of a patient with advanced HCC who developed cutaneous, pulmonary, and lymphatic metastases following failure of multiple lines of tyrosine kinase inhibitors (TKIs) including donafenib and regorafenib, and lenvatinib plus anti-programmed death-1 (PD-1) monoclonal antibody (tislelizumab) alongside radiotherapy. Next-generation sequencing (NGS) of the primary liver surgical sample confirmed wild-type EGFR status without canonical driver mutations, whereas retrospective immunohistochemistry (IHC) and cyclic multiplexed tyramide signal amplification (CmTSA) of the cutaneous metastatic sample demonstrated EGFR protein overexpression. Subsequent spatial analysis demonstrated that EGFR-high tumor regions strongly correlated with hyper-proliferative Ki67+ tumor cells. Furthermore, this EGFR-high niche was spatially associated with an immunosuppressive microenvironment characterized by the infiltration of PD-L1+ myeloid cells, CD14+ monocytes, regulatory T cells (Tregs), and PD-L1+/IDO1+ tumor cells. Therefore, we formulated a spatial profiling-guided therapeutic hypothesis and added gefitinib to the prior regimen of lenvatinib plus tislelizumab concurrent with radiotherapy, achieving significant tumor regression despite subsequent intracranial progression. In conclusion, these findings suggest that spatial profiling offers valuable therapeutic insights, highlighting the potential benefit of an EGFR-targeted triplet strategy in refractory HCC with EGFR protein overexpression. While confounding effects from concurrent therapies cannot be excluded, this profiling-guided hypothesis warrants further prospective clinical and mechanistic validation.

## Introduction

Current systemic treatments for advanced hepatocellular carcinoma (HCC) mainly include anti-angiogenesis targeted therapy combined with immune checkpoint inhibitor (ICI) immunotherapy, dual immunotherapy, or targeted and immunotherapeutic monotherapies (1). Before the era of immunotherapy for HCC, sorafenib and lenvatinib were commonly used as first-line therapies (2, 3). In China, donafenib has also been approved as a first-line indication for HCC, with a better overall survival (OS) than sorafenib in the phase II/III clinical trial (ZGDH3) (4). Subsequent three global phase III clinical trials showed that compared to sorafenib or sorafenib/lenvatinib, atezolizumab plus bevacizumab (IMbrave150) (5), durvalumab plus tremelimumab (HIMALAYA) (6), and nivolumab plus ipilimumab (CheckMate 9DW) (7) resulted in significantly better OS and objective response rate (ORR). Although the global LEAP-002 trial failed to reach its primary endpoints with lenvatinib plus pembrolizumab compared to lenvatinib, the combined regimen demonstrated a further extended gap of mOS with 3.4 months in Asian populations with more hepatitis B virus (HBV)-related HCC (8). In the real world of China, lenvatinib plus PD-1 monoclonal antibody or combined with transarterial chemoembolization (TACE) therapy was often used (9). In the CARES-310 trial, rivoceranib plus camrelizumab showed significant benefit in PFS and OS compared with sorafenib (10). Sequential targeted therapy or a switch to immunotherapy has been approved as second-line treatment after sorafenib failure. However, there is no phase III trial supporting second-line options after the failure of the other new first-line treatment except for sorafenib. For patients who have previously experienced tyrosine kinase inhibitors (TKI) and immunotherapy failure, clinical treatment also faces challenges.

Drug resistance in HCC arises from multifaceted causes and sophisticated mechanisms. Mechanisms of resistance to TKI therapy frequently arise from activated pivotal oncogenic cascades like the RAS/RAF/MEK/ERK signaling pathway, alongside epigenetic dysregulation (e.g., aberrant DNA/RNA methylation), the infiltration of immunosuppressive cells within the tumor microenvironment (TME), and metabolic reprogramming (11). Regarding ICI therapy, resistance mechanisms primarily encompass defects in antigen presentation, the upregulation of alternative immune checkpoints, the formation of an immunosuppressive environment [via indoleamine 2,3-dioxygenase (IDO) and adenosine accumulation], and gut dysbiosis (12). Elucidating these mechanisms is important for overcoming refractory HCC. Recent advances in spatially resolved single-cell omics have linked spatial features to clinical outcomes. Therefore, spatial profiling based on formalin-fixed paraffin-embedded (FFPE) tissue can be used as a promising translational platform to uncover potential targetable resistance hubs and implement personalized strategies.

Herein, we report a refractory HCC patient harboring cutaneous metastases who experienced rapid disease progression after failure of various lines of therapies. Through TME analysis of the metastatic cutaneous sample, we observed a potential association between EGFR overexpression, tumor cell proliferation, and an immunosuppressive microenvironment, which informed a hypothesis-generating model that guided our EGFR-targeted salvage strategy (**Figure 1A**).

**Figure 1:**
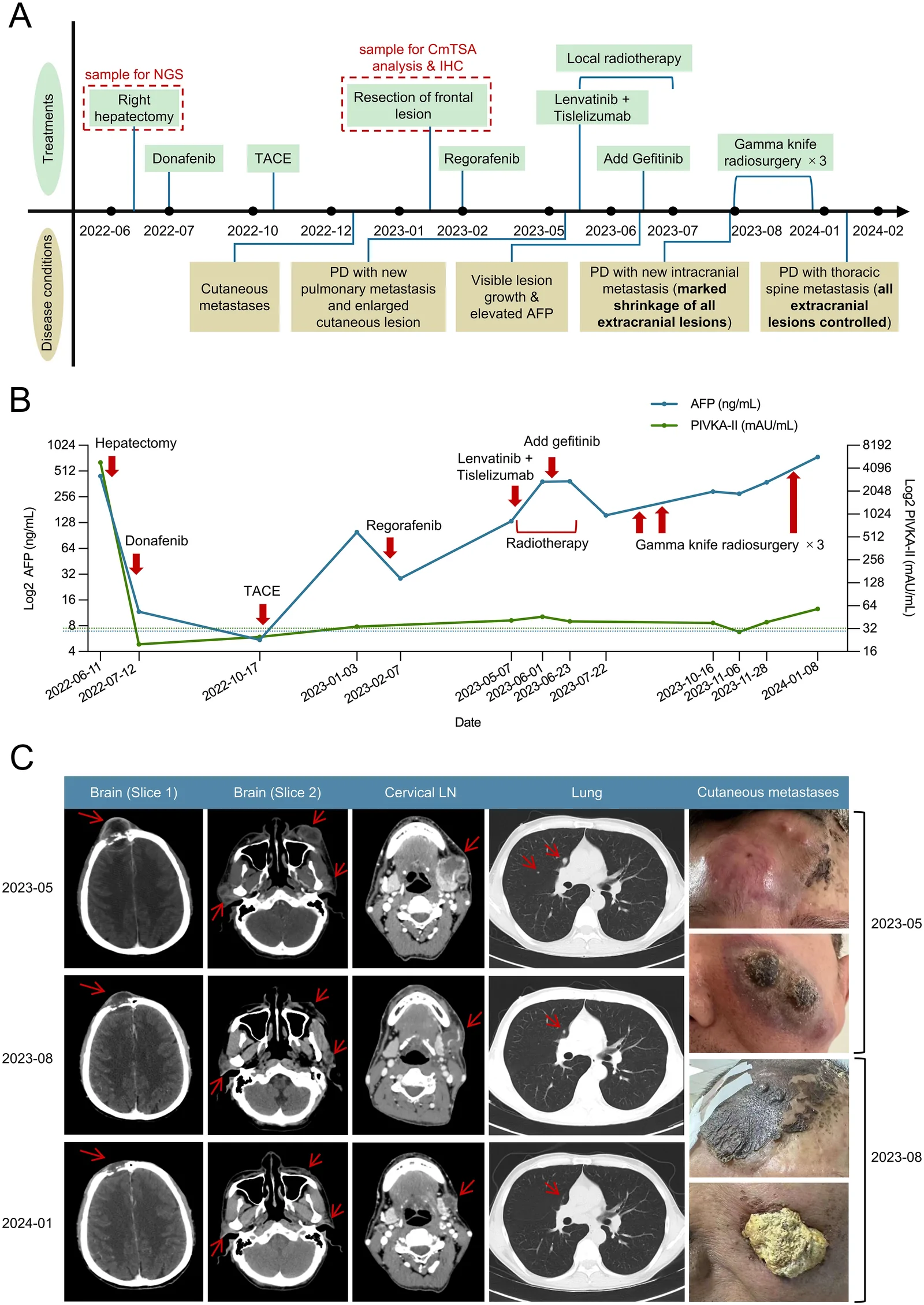
The treatment course and therapeutic outcome of the HCC metastatic patient. **(A)** Schematic overview of the therapeutic timeline, including surgical interventions, sequential systemic treatments, and key clinical timing. Red dashed boxes indicate the time points for NGS, IHC and spatial profiling (CmTSA) sample collection. **(B)** Longitudinal monitoring of serum AFP and PIVKA-II levels throughout the treatment course. The horizontal dashed lines indicate the upper limit of normal (ULN) for AFP and PIVKA-II, respectively. **(C)** Radiologic and clinical evidence of tumor shrinkage across all extracranial lesions (including the right frontal region, left maxillofacial region, bilateral parotid regions, cervical lymph node, and pulmonary nodules) following the administration of gefitinib. Red arrows highlight the lesions.

## Case presentation

A 47-year-old male with a history of hepatitis C virus–related cirrhosis was found to have a hepatic mass during a routine physical examination. Contrast-enhanced computed tomography (CT) of the abdomen revealed a 5.6 × 5.8 cm hypodense lesion in the right hepatic lobe. Laboratory tests showed an elevated serum alpha-fetoprotein level (AFP, 448 ng/mL). The patient underwent a right hepatectomy in June 2022. Pathology confirmed poorly to moderately differentiated hepatocellular carcinoma (HCC) with microvascular invasion (MVI). Subsequent next-generation sequencing (NGS; 341-gene panel) of this specimen identified TP53 (p.Y220C) and TERT promoter mutations, along with CCND1/FGF19 and MCL1/NTRK1 co-amplification, and confirmed a wild-type EGFR status without canonical activating mutations. Postoperatively, the patient was initiated on the oral TKI donafenib (200 mg twice daily) in July 2022, followed by a single session of TACE in October 2022. Two months later, multiple cutaneous nodules on the head and face were found, with the patient maintaining an Eastern Cooperative Oncology Group performance status (ECOG PS) of 0. Biopsy confirmed cutaneous metastases of HCC (Barcelona Clinic Liver Cancer [BCLC] stage C). Positron emission tomography/computed tomography (PET/CT) revealed multiple metastatic lesions in the head, face, and neck, with no evidence of recurrence in the remnant liver. In January 2023, the patient underwent resection of the frontal cutaneous tumor. He then received regorafenib (160 mg once daily) from February to May 2023. Follow-up enhanced CT in May 2023 confirmed progressive disease (PD) according to the Response Evaluation Criteria in Solid Tumors (RECIST) version 1.1 (13), evidenced by the enlargement of the cutaneous metastases and the emergence of new pulmonary metastases (**Figure 1C**). The AFP level progressively increased from 28.5 ng/mL to 133 ng/mL. In May 2023, the treatment was switched to a combination of lenvatinib (8 mg once daily) plus tislelizumab (200 mg every 3 weeks), concurrent with localized radiotherapy. The radiotherapy was delivered in two sequential phases: phase I consisted of intensity-modulated radiation therapy (IMRT, 40 Gy in 20 fractions) targeting the left maxillofacial, frontal, bilateral parotid regions and cervical lymph node, followed by a phase II boost (21 Gy in 7 fractions) utilizing volumetric modulated arc therapy with image-guided radiation therapy (VMAT-IGRT) to the head and neck target volume. However, after one month, this regimen also failed to halt the visible enlargement of metastases or the elevation of the AFP level(from 133 ng/mL to 388 ng/mL; **Figure 1B**).

## Panoramic profiling of the tumor immune microenvironment

For the aforementioned reason, we performed in-depth histological and microenvironmental analysis of the frontal cutaneous metastasis resected in January 2023. We utilized cyclic multiplexed tyramide signal amplification (CmTSA) staining with a comprehensive 21-biomarker panel on the FFPE sections from the patient (Detailed information can be found in Doc S1). The analysis identified and segmented 22 distinct cell types including various subtypes of PD-L1+ tumor cells (PD-L1+ TCs), IDO1+ tumor cells (IDO1+ TCs), lymphoid cells and others (**Figure 2C**). Subsequently, a single-cell and spatially resolved map of the TME was constructed.

**Figure 2:**
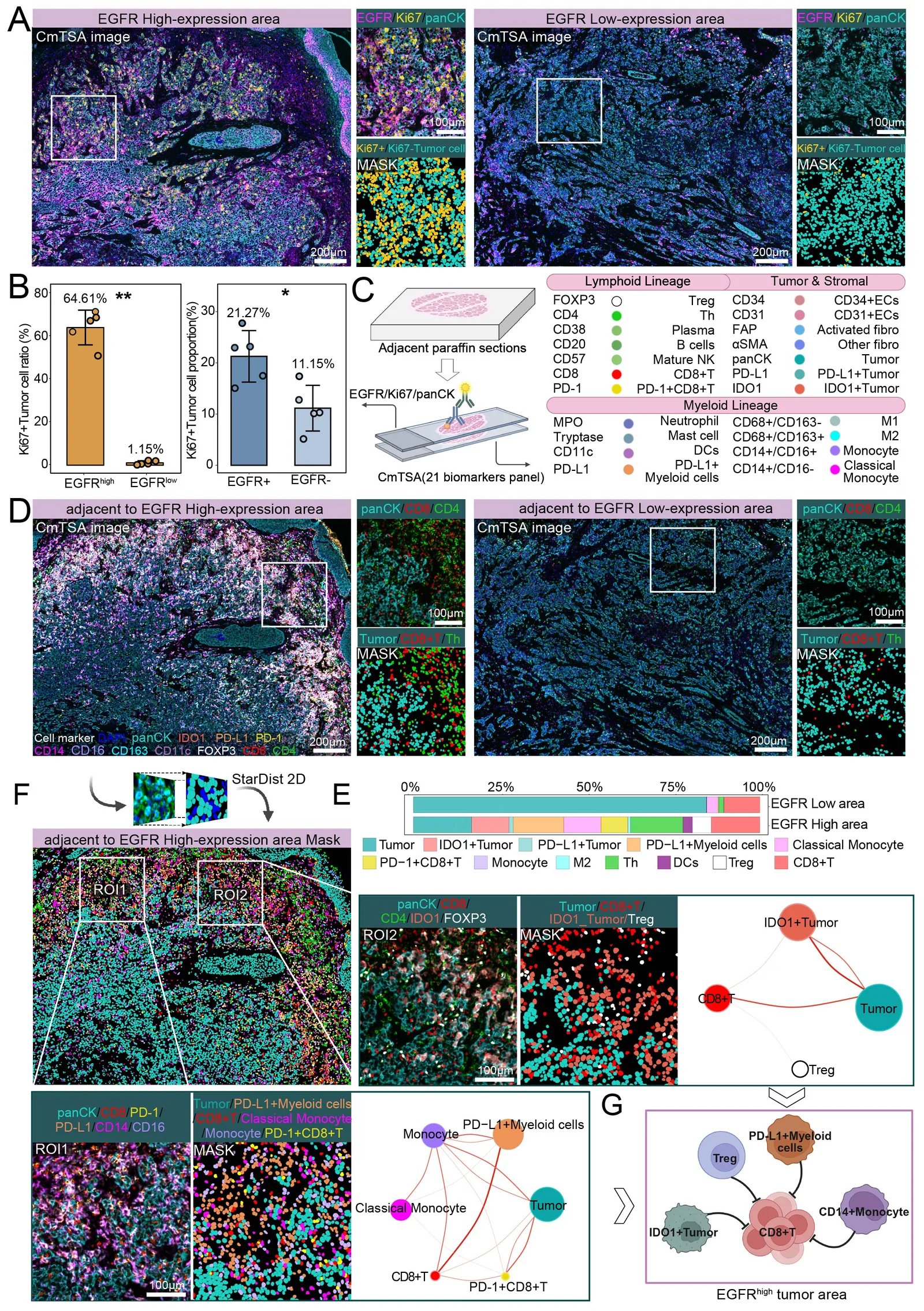
Dissection of the tumor immune microenvironment features in the cutaneous metastasis resected in January 2023. **(A)** CmTSA staining (EGFR/Ki67/panCK) shows the heterogeneous expression patterns of EGFR and Ki67; a cell annotation mask was generated using the StarDist 2D nuclear segmentation algorithm; areas within the white boxes are magnified on the right. **(B)** Left: Statistical comparison of the ratio of Ki67+ tumor cells between EGFR-high and EGFR-low regions. Each dot represents one randomly selected sub-ROI (n=5 per group), with bars indicating the mean ± SD (EGFR-high: 64.61% ± 8.14%; EGFR-low: 1.15% ± 0.92%). (Mann-Whitney U test, r = 0.79, *p* = 0.008); right: Comparison of Ki67^+^ proportions between EGFR^+^ and EGFR^-^ tumor cells in EGFR-high region. Each pair of connected dots represents the two subsets measured from the same ROI (n = 5 paired ROIs). Bars indicate mean ± SD: EGFR^+^Ki67^+^, 21.3% ± 5.03%; EGFR^-^Ki67^+^, 11.1% ± 4.46% (paired t-test, d = 1.59, *p* = 0.024). The paired design accounts for spatial matching of the two cell subsets within each ROI. All data are presented as mean ± SD. **(C)** An adjacent paraffin section was subjected to CmTSA staining with a 21-biomarker panel to profile immune-related cells; using StarDist 2D and biomarker combinations, a total of 22 cell types were annotated. **(D)** CmTSA staining (21-biomarker) of an adjacent paraffin section shows the significant difference in distribution of immune-related cells between EGFR-high (left) and EGFR-low (right) tumor areas; a cell annotation mask was generated using StarDist 2D; areas within the white boxes are magnified on the right. **(E)** Stacked bar plot showing the proportions of immune-related cell types located in EGFR-high and EGFR-low tumor areas. **(F)** Analysis of cell–cell interactions among immune-related cells in the EGFR-high tumor area; bottom left (ROI1) shows intensive interactions of PD-L1+ myeloid cells and monocytes with CD8+ T cells; middle right (ROI2) shows intensive interactions of IDO1+ tumor cells and Tregs with CD8+ T cells. **(G)** Schematic illustration of the potential multiple immunosuppressive effects on CD8+ T cells within the EGFR-high tumor area.

Multiplex immunofluorescence co-staining for EGFR and Ki67 revealed a spatial association between EGFR expression and tumor cell proliferation. Quantitative analysis demonstrated that the proportion of Ki67+ tumor cells was significantly higher in EGFR-high regions compared to EGFR-low regions (64.61% ± 8.14% vs. 1.15% ± 0.92%; r = 0.79; **Figures 2A, B**). Furthermore, the percentage of proliferating (Ki67+) cells was significantly higher within the EGFR+ tumor cell population than in the EGFR-negative population (21.27% ± 5.03% vs. 11.15% ± 4.46%; d = 1.59). Together, these findings indicate a spatial association between EGFR expression and increased tumor cell proliferation within this metastatic lesion, suggesting that EGFR-high regions may represent areas with enhanced proliferative activity.

Spatial analysis further indicated that EGFR-high regions were also associated with an immunosuppressive landscape. Specifically, quantification revealed an enrichment of PD-L1+ and IDO1+ tumor cells, PD-L1+ myeloid cells, CD14+ monocytes, and regulatory T cells (Tregs) in regions adjacent to EGFR-high tumor cells compared to EGFR-low regions (**Figures 2D, E**). Neighborhood analysis revealed specialized niches uniquely localized within EGFR-high areas. In the first region of interest (ROI1), CD8+ cytotoxic T lymphocytes (CTLs) penetrated the tumor periphery, spatially co-localizing with densely clustered PD-L1+ myeloid cells. Additionally, an abundance of proximal undifferentiated monocytes was noted in these regions, which warrants further investigation regarding their potential role in myeloid polarization (**Figure 2F**). In ROI2, a close spatial proximity among IDO1+ TCs, forkhead box P3+ (FOXP3+) Tregs, and CTLs was observed at the invasive front (**Figure 2F**). In summary, we synthesized these findings into a schematic model (**Figure 2G**), illustrating the formation of a distinct immunosuppressive niche adjacent to EGFR-high regions. This model illustrates that the spatial aggregation of inhibitory immune populations may contribute to the local suppression of CD8+ T cell activity and therapeutic resistance.

Collectively, while prior NGS of the primary liver specimen confirmed wild-type EGFR status without canonical driver mutations, further immunohistochemistry (IHC) testing of the cutaneous metastasis revealed membranous positivity for the EGFR protein (score: 2+). These integrated molecular and spatial profiles delineate a proliferative and immunosuppressive tumor architecture associated with EGFR overexpression, thereby providing a basis for our subsequent hypothesis regarding a potential compensatory mechanism.

## Rationale for therapeutic intervention and treatment design

Based on our spatial profiling results, we observed an intricate TME profile associated with EGFR overexpression, characterized by two distinct but potentially interconnected compartments: (1) an intrinsic highly proliferative state (indicated by EGFR+/Ki67+ co-localization), and (2) an extrinsic immunosuppressive niche driven by the spatial aggregation of PD-L1+/IDO1+ TCs, monocytes, and Tregs. This localized architecture informed a hypothesis-generating mechanistic model (**Figure 3**), which offered a potential rationale to consider a multi-target salvage strategy. Consequently, due to the visible enlargement of metastases and the elevation of AFP levels, a novel triplet therapy combining the EGFR inhibitor gefitinib (250 mg once daily), lenvatinib (8 mg once daily) and tislelizumab (200 mg every 3 weeks) was administered in June 2023.

**Figure 3:**
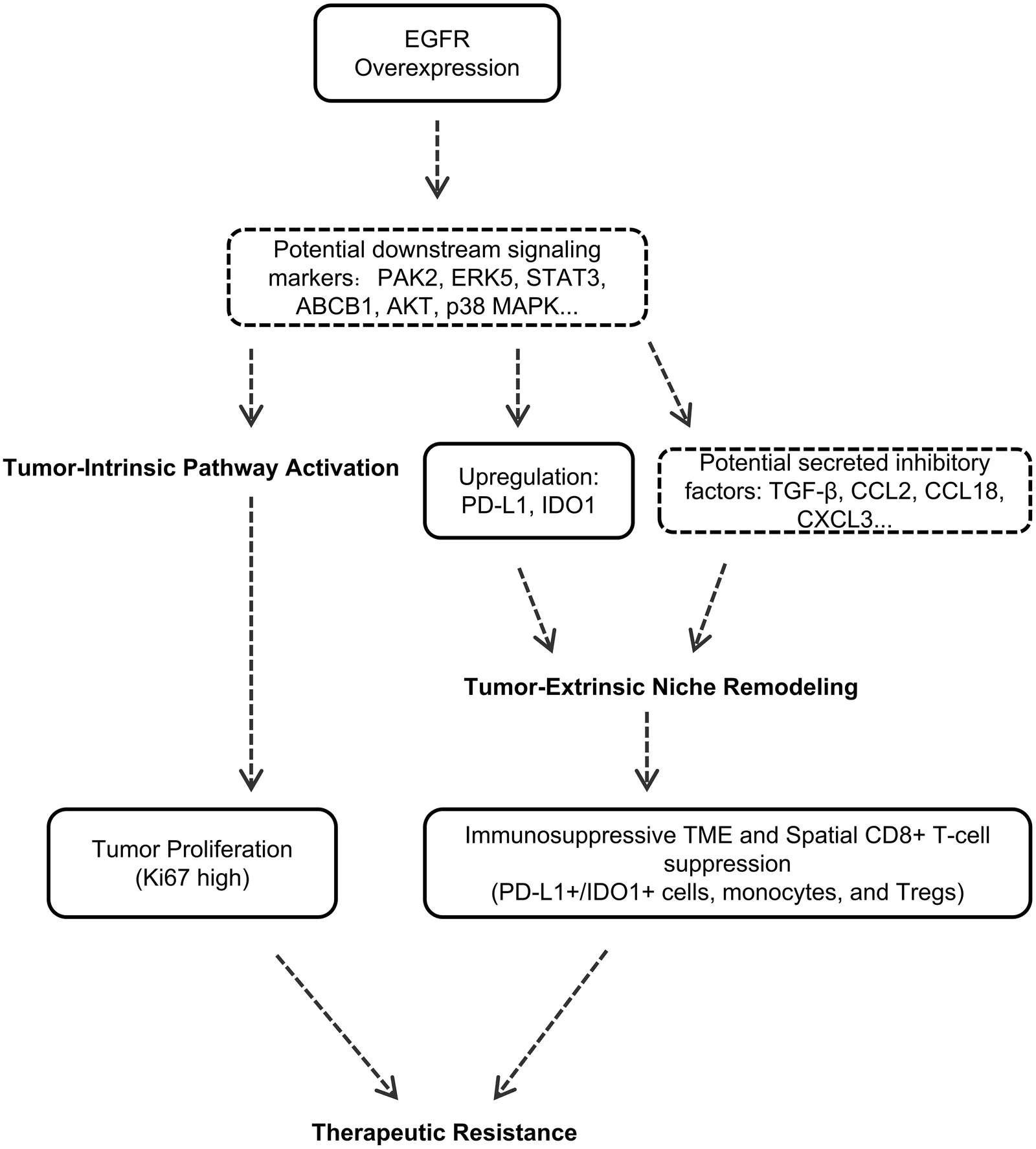
A simplified schematic of the hypothetical resistance mechanism based on spatial analysis and existing literature. Solid-line boxes represent biological entities and spatial phenotypes directly measured in this study via IHC and multiplex spatial profiling (e.g., EGFR overexpression, Ki67 proliferation, PD-L1/IDO1 upregulation, and spatial CD8+ T-cell suppression). Dashed-line boxes denote downstream signaling mediators and secreted inhibitory factors inferred from existing literature. Dashed arrows indicate hypothesized mechanistic cascades and functional connections rather than experimentally validated causal steps in this cohort.

## Treatment outcomes and clinical follow-up

After more than one month with the triplet therapy, alongside continued radiotherapy (administered from May 2023 to July 2023), the cutaneous, lung and lymph node metastatic lesions obtained marked shrinkage, with the AFP level decreasing sharply from 388 ng/mL to 156 ng/mL (**Figure 1**). In August 2023, localized intracranial metastases emerged and were successfully managed with gamma knife radiosurgery. Despite an overall response assessment of PD according to the RECIST version 1.1, the patient achieved a remarkable and durable response in all extracranial lesions (including the right frontal region, left maxillofacial region, bilateral parotid regions, cervical lymph node, and pulmonary nodules) (**Figure 1C**). Concurrently, the patient continued the triplet therapy. From June 2023 to January 2024, no new extracranial lesions were detected. In January 2024, the patient developed PD with a new thoracic spinal metastasis, while all original extracranial lesions remained well-controlled. He subsequently returned to his local hospital and was lost to follow-up. Given that the blood-brain barrier remains a clinical challenge, the overall PFS was nearly 2 months, whereas the duration of extracranial disease control approached nearly 7 months until a thoracic spinal metastasis occurred. Throughout the entire treatment course, the patient maintained well-preserved liver function (Child-Pugh Class A), and no grade 4 or 5 treatment-related adverse events (AEs) occurred. The only grade 3 AE was thrombocytopenia, which was related to liver cirrhosis and was alleviated via pharmacological intervention, underscoring the tolerable safety of this intensive salvage approach.

## Discussion

Extrahepatic metastases of HCC predominantly involve the lungs, bones, and lymph nodes (14). Cutaneous metastasis is a relatively rare presentation (2.7%–3.4%) that typically indicates an aggressive, rapidly progressive phenotype with a poor prognosis (15). We report a case of refractory HCC with cutaneous metastasis exhibiting a remarkable extracranial response to a personalized combination therapy (gefitinib, lenvatinib, tislelizumab, and local radiotherapy). Interpreting the clinical response in this complex case requires careful analysis. Notably, after one month of concurrent lenvatinib, tislelizumab, and local radiotherapy, we observed visible enlargement of the cutaneous metastases and elevated tumor markers. The subsequent addition of gefitinib to this regimen was followed by marked and durable regression not only in the irradiated cutaneous lesions but also in distant, non-irradiated sites, such as pulmonary nodules. While this sequential timeline suggests a potential therapeutic contribution from gefitinib, definitive causal attribution cannot be established from a single case. Alternative explanations must be rigorously considered, including spontaneous disease fluctuations, delayed responses to immunotherapy, or the abscopal effect—a phenomenon defined as systemic anti-tumor immunity triggered by local radiotherapy at distant, non-irradiated sites, primarily driven by immunogenic cell death that activates dendritic cells to prime CD8+ T-cell responses (16). Additionally, the clinical evaluation must account for site-specific variations. The emergence of intracranial metastases may stem from the limited penetration of systemic agents across the blood-brain barrier. Following gamma-knife radiosurgery for intracranial control, the patient maintained profound disease control until the development of bone metastasis. Ultimately, based on our microenvironmental findings, this gefitinib-based combination therapy represents a potentially effective strategy that warrants further investigation and validation through both clinical and basic research.

In non-small cell lung cancer (NSCLC), the clinical efficacy of EGFR-targeted TKIs is typically tied to activating mutations. Conversely, classical activating mutations in the EGFR kinase domain are exceptionally rare in HCC, with previous small-scale studies failing to detect them (17, 18). However, EGFR overexpression is frequently observed and strongly correlates with tumor aggressiveness, metastasis, and resistance (17). While the clinical application of these inhibitors in those with wild-type EGFR remains highly controversial, Wu et al. demonstrated that gefitinib yielded comparable response rates and survival outcomes regardless of EGFR mutational status (19). In our case, NGS was limited to the primary liver sample, while the cutaneous metastasis was assessed only for protein expression (via IHC and CmTSA). Although secondary genomic alterations in the metastasis cannot be completely excluded, our therapeutic rationale was primarily grounded in the observed EGFR protein overexpression. Mechanistically, EGFR inhibitors such as gefitinib suppress ATP binding and downstream PI3K/AKT and MEK/ERK signaling in HCC subpopulations with EGFR overexpression, ultimately triggering cell cycle arrest, apoptosis, and antimetastatic effects (20).

Targeting these pathways is particularly critical for overcoming therapeutic resistance. Notably, preclinical models have extensively characterized EGFR’s role in driving drug resistance to first-line targeted agents. In HCC, lenvatinib-resistant cells escape therapeutic efficacy via feedback activation of the EGFR–PAK2–ERK5 axis, a pathway that can be effectively blocked by the addition of EGFR inhibitors like gefitinib (21). Moreover, the EGFR–STAT3–ABCB1 axis also has been shown to restore intrinsic proliferative capacity and promote drug efflux (22). Building upon these preclinical foundations, a prospective, single-arm clinical trial has demonstrated the efficacy and safety of combining lenvatinib with gefitinib in patients with lenvatinib-resistant, EGFR-high HCC, achieving an encouraging ORR of 30% (23). These studies collectively underscore EGFR hyperactivation in HCC as a central hub for tumor survival and therapeutic escape, which can be robustly blocked by EGFR inhibitor combinations (20). Beyond driving intrinsic tumor survival, established paradigms in various other solid tumors indicate that activated EGFR signaling orchestrates an immunosuppressive TME by upregulating immune checkpoints like PD-L1 and secreting inhibitory factors (e.g., TGF-β, CCL2, CCL18, CXCL3) that impair T-cell infiltration and cytotoxicity while recruiting tumor-associated macrophages (TAMs) and Tregs (24). Studies have shown that EGFR activation through the AKT-signal transducer and activator of transcription 3 (STAT3) pathway or the EGFR–p38 mitogen-activated protein kinase (MAPK) axis drives PD-L1 expression (25–29). Similarly, in lung cancer, IDO1 exerts potent immunosuppressive effects by depleting CD8^+^ T-cell infiltration and upregulating Treg function in significant correlation with EGFR status (30). Based on previous studies, we hypothesized that EGFR overexpression may act as a pivotal hub associated with both intrinsic tumor proliferation and an immunosuppressive microenvironment. Specifically, this sustained EGFR signaling—potentially through the MAPK, STAT, or ERK5 axes— might contribute to the observed PD-L1 and IDO1 upregulation at the invasive front, and promote the secretion of inhibitory factors like TGF-β and CCL2. These factors may facilitate monocyte recruitment, inhibitory myeloid polarization, and Treg infiltration, thereby spatially suppressing CD8+ T cells and driving therapeutic resistance (**Figure 3**). Importantly, the specific signaling pathways detailed in our model are partly extrapolated from non-HCC preclinical studies and warrant further validation in HCC. As functional assays were not performed on this patient’s samples, these pathways represent a hypothesized rationale for gefitinib’s potential efficacy rather than definitive causal mechanisms.

Most clinical trials evaluating ICIs in HCC fail to meet their endpoints due to complex combination challenges and resistance within the highly immunosuppressive TME. This underscores an urgent clinical need for biomarker-driven strategies (31). Beyond clinical efficacy, this case exemplifies a shift from empirical drug treatment toward spatially informed precision oncology. We identified a distinct architectural signature of immunosuppression at the tumor’s invasive front—characterized by the spatial convergence of inhibitory myeloid cells and checkpoint-rich malignant clusters. Such high-resolution analysis is critical for deconstructing resistance landscapes and identifying targetable hubs, like EGFR, to optimize future combination therapies.

This study has several limitations. First, the observed clinical benefit cannot be definitively attributed to the independent effect of gefitinib; individual variability may influence the response to this EGFR-targeted combination therapy, and we cannot exclude potential abscopal effects from concurrent radiotherapy. Consequently, the generalizability of these findings is limited. Second, our spatial profiling findings reflect observational associations rather than definitive causal mechanisms. This limitation is compounded by the exploratory nature of the methodology, the restricted number of analyzed regions of interest (n = 5 ROIs per group) despite the large observed effect sizes, the absence of paired pre- and post-treatment specimens for comparison, and the potential observer and sampling bias associated with manual ROI selection. Finally, our outcomes reflect organ-specific disease control rather than an overall partial response. Larger prospective studies with rigorous cohorts are necessary to validate these spatial and mechanistic findings and to determine the prevalence and clinical significance of EGFR overexpression in advanced HCC.

Overall, this case highlights the utility of spatial TME profiling in guiding salvage therapy for refractory HCC. Furthermore, our findings suggest that EGFR overexpression may characterize a distinct but infrequent subgroup of HCC patients for whom combined EGFR-targeted interventions could be beneficial. These observations, however, remain preliminary, warranting further prospective validation in larger cohorts and in-depth mechanistic studies to confirm their clinical applicability.
