# Supplementary material for: Gefitinib-based therapy in refractory hepatocellular carcinoma with EGFR overexpression: a spatial profiling-guided case report
Source: Front Immunol. 2026 Sep 24;17:1891806. doi: 10.3389/fimmu.2026.1891806 (PMC13655545; doi:10.3389/fimmu.2026.1891806)
Supplement: Supplementary file 1 [file DataSheet1.pdf]

MATERIALS AND METHODS

Cyclic multiplexed Tyramide Signal Amplification (CmTSA) and analysis strategy

(1) Collection of clinical samples

FFPE sections were obtained from the Department of Pathology, West China Hospital, Sichuan University. Viable tissue samples were collected during the patient's clinical treatment. Participants gave informed consent.

H&E and IHC results

Staining and image acquisition were completed by the Department of Pathology, West China Hospital, Sichuan University.

(2) Antibody information

Table S1. Antibody information

Antibody	Brand	Catalog#	Dilution rate
FOXP3	HUABIO	IRS053	1:100
CD57	HUABIO	IRS027	1:100
MPO	HUABIO	IRS037	1:100
CD4	HUABIO	IRS005	1:100
Tryptase	HUABIO	IRS041	1:100
CD11c	HUABIO	IRS001	1:100
CD163	HUABIO	IRS002	1:100
CD68	HUABIO	IRS004	1:100
CD14	HUABIO	IRS015	1:100
CD16	HUABIO	IRS017	1:100
CD38	HUABIO	IRS006	1:100
CD20	HUABIO	IRS003	1:100
CD34	HUABIO	IRS024	1:100
CD31	HUABIO	IRS023	1:100
CD8	Immunoway	YM6938	1:500
PD1	ZSGB-BIO	ZM-0381	1:800
FAP	zenbio	R381838	1:1000
α-SMA	HUABIO	IRS012	1:100
panCK	HUABIO	IRS010	1:100
Ki67	HUABIO	IRS036	1:100
IDO1	HUABIO	IRS045	1:100
PD-L1	abcam	ab228462	1:1000
EGFR	HUABIO	IRS046	1:100

(3) CmTSA staining

Initially, CmTSA staining was performed using IRISKit ® HyperView Quench Buffer (#MH900102) for background fluorescence and specific staining light quenching tissue processing in LUMINIRIS Fluorescence Quenching System (#MH030101). Following this, fluorescent staining for antibody labeling was performed using the IRISKit™ HyperView mTSA Kit (#MH900206). Image acquisition was completed in an Evident Whole Slide Scanning System VS200. Then, multiple rounds of images were aligned using IRRISSin, resulting in super-multiplexed metric staining of a single section.

(4) CmTSA image analysis

Delineation of Tumor and Normal regions

Through image observation, we found that there were obvious boundaries between tumor and normal regions as well as differences in the density of immune cells, so the corresponding CmTSA images were delineated into Tumor and Normal regions under the supervision of pathologists.

Definition and delineation of EGFR-high and EGFR-low regions

The viable tumor compartment was first confirmed by a pathologist with reference to H&E morphology and panCK staining. The whole-slide image was then reviewed under identical acquisition and display settings to assess the spatial heterogeneity of membranous EGFR fluorescence. During region delineation, only the EGFR and DAPI channels were displayed, whereas the Ki67 and immune-marker channels were concealed. ROI selection was therefore performed while blinded to the spatial distributions of the downstream proliferation and immune microenvironment variables. DAPI was used only for nuclear identification, cell segmentation, and tissue orientation and was not used to classify EGFR expression.

A spatially continuous tumor area showing relatively intense membranous EGFR fluorescence was manually delineated as the relative EGFR-high parent region, whereas a viable tumor area showing relatively weak membranous EGFR fluorescence was delineated as the relative EGFR-low parent region. Necrotic or hemorrhagic areas, tissue folds, section edges, scanning artifacts, and regions with prominent nonspecific fluorescence were excluded. For quantitative comparisons, five identically sized, non-overlapping sub-ROIs measuring 400 μm × 400 μm were randomly selected according to the predefined eligibility criteria from each parent region. Each sub-ROI contained viable panCK-positive tumor tissue and satisfied the same exclusion criteria. The ten sub-ROIs were treated as within-specimen spatial sampling units rather than independent biological replicates.

Cell segmentation was performed using the StarDist-2D algorithm implemented in QuPath. The mean membranous EGFR fluorescence intensity was extracted from segmented panCK-positive tumor cells. The whole-specimen median EGFR membrane mean intensity of 67.5 was used as a post-selection quantitative reference and not as a prespecified threshold for parent-region delineation. As a post-selection consistency check, 75.53% of panCK-positive tumor cells in the relative EGFR-high region exhibited membrane EGFR intensity above the whole-specimen median of 67.5, whereas 59.22% of tumor cells in the relative EGFR-low region exhibited values below the median. Accordingly, the two parent regions were interpreted as relative spatial expression patterns within the same specimen rather than as biologically discrete EGFR categories.

Cell segmentation and positive threshold setting

The pretrained StarDist-2D model was implemented in QuPath (v0.5.1) to segment DAPI-positive nuclei and generate cell-level objects in the multiplex fluorescence images. For the original cell-classification analysis, the mean fluorescence intensity measured within the DAPI-derived nuclear segmentation mask (Nucleus: Mean) was used as the common analytical feature for all marker channels.

Following registration, fluorescence channels were analyzed using a uniform 8-bit intensity range of 0–255. Each marker channel was reviewed separately in representative regions containing clearly negative background, weakly positive signals, and strongly positive signals. A

marker-specific positivity threshold was then manually selected within the 0–255 range. For each segmented cell, fluorescence intensity was extracted using the standardized intensity feature generated by the segmentation pipeline. Although nuclear segmentation was used as the cellular reference framework, marker positivity was determined based on the marker-specific fluorescence signal obtained from the registered single-cell objects rather than the biological localization of the protein itself.

Threshold classifications were visually verified by overlaying predefined colored masks on the original single-channel fluorescence images. Tissue folds, section edges, scanning artifacts, autofluorescence, and regions with prominent nonspecific background were excluded during quality control. Once finalized, the same marker-specific threshold was applied throughout the corresponding whole-slide image and was not adjusted separately among different ROIs or according to downstream spatial-analysis results. Then the x- and y-coordinates of all segmented cells were subsequently exported for spatial analysis.

Marker-guided cell phenotyping and lineage assignment

Following cell segmentation and marker-positivity determination, cellular phenotypes were assigned using a supervised, marker-combination–guided cell-gating framework adapted from the CmTSA spatial profiling pipeline described by Xiao et al[1]. Cell identities were annotated according to characteristic lineage-marker expression, marker co-expression patterns, and staining-quality supervision.

Populations represented by a characteristic lineage marker were assigned according to the corresponding marker-positive phenotype, whereas populations requiring multiple markers were identified according to their combined expression patterns. Expected population abundance was used only as a post-annotation plausibility check to identify potential staining or classification abnormalities and was not used to adjust marker-positivity thresholds or force phenotype frequencies. The complete marker-combination scheme used to annotate the 22 marker-defined cell populations is shown in Figure S1.

Table S2. Marker-specific fluorescence intensity thresholds used for single-cell positivity classification

Marker	Positive definition (Nucleus:Mean ≥)	Marker	Positive definition (Nucleus:Mean ≥)
FOXP3	18	CD34	15
CD57	20	CD31	20
MPO	30	CD8	26
CD4	20	PD1	21
Tryptase	20	FAP	25
CD11c	20	α-SMA	25
CD163	8	panCK	14
CD68	16	Ki67	20
CD14	14	IDO1	24
CD16	10	PD-L1	11
CD38	30	EGFR	30
CD20	18		

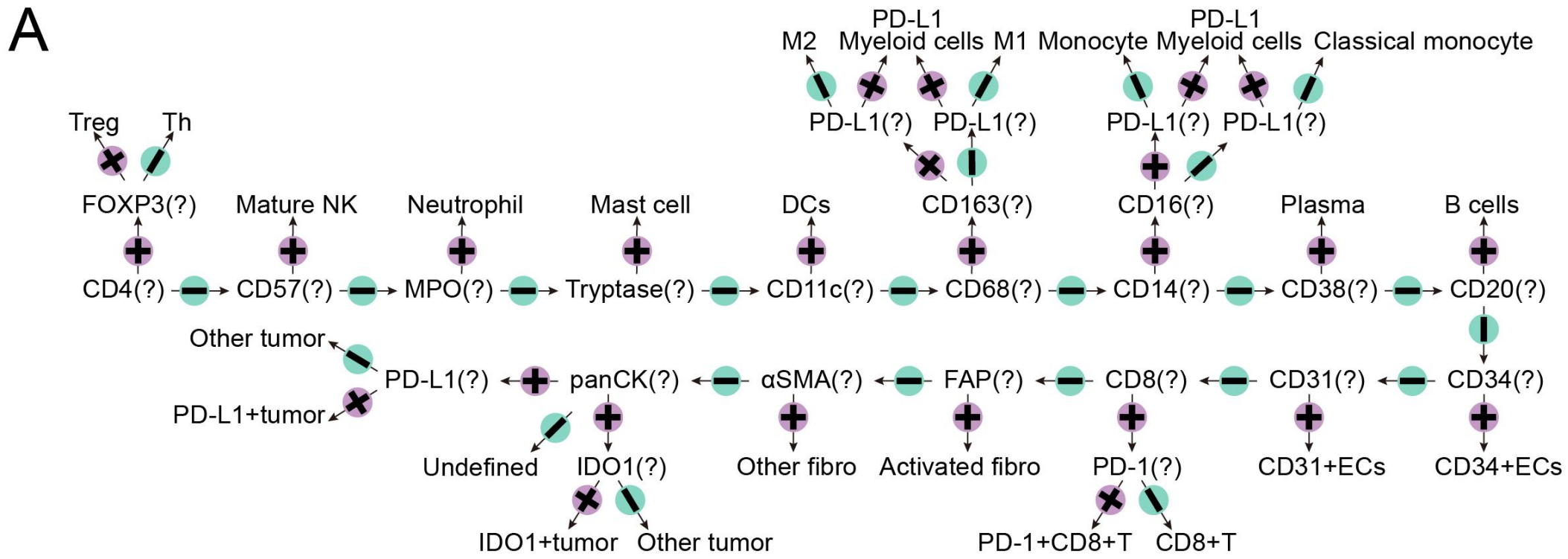

Figure S1. Marker-combination–guided annotation scheme for the 22 cell populations identified using the 21-marker CmTSA panel.

(5) Spatial neighborhood and cell–cell interaction analysis

Single-cell spatial coordinates derived from QuPath were imported into R version 4.3.1. Spatial neighborhood analysis was performed using an adapted implementation of the SOAPy workflow described by Xiao et al[1]. A fixed neighborhood radius of 100 μm was applied. Two cells were defined as spatial neighbors when the Euclidean distance between their centroids was ≤100 μm, and each neighboring cell pair was represented as an undirected edge in the spatial network. Each cell pair was counted once. For each pair of annotated cell phenotypes, the interaction metric was derived from the observed number of edges directly connecting the two phenotypes and was normalized relative to the numbers of edges connecting either phenotype to all other cells. This normalization was used to reduce the influence of differences in cell abundance and overall connectivity.

Statistical assessment was performed using 100 permutations to generate the expected interaction distribution under a randomized cell-type configuration while preserving the observed tissue geometry and cell numbers. The observed interaction metric was standardized against the permutation-derived mean and standard deviation to obtain a Z score. A positive Z score indicated spatial proximity greater than expected by chance, whereas a negative Z score indicated relative spatial separation. These analyses were exploratory and were used to characterize within-specimen spatial organization. All spatial analyses were conducted within a single specimen.

(6) Statistical analysis

All statistical analyses were performed using R version 4.3.1. Continuous ROI-level summaries are presented as mean ± standard deviation. For the comparison of Ki67-positive tumor-cell proportions between EGFR-high and EGFR-low regions, five sub-ROIs were evaluated per region. ROI-level proportions were summarized descriptively, and statistical significance was evaluated using the Mann–Whitney U test. The corresponding nonparametric effect size was quantified using rank-biserial correlation (r), calculated from the Mann–Whitney U statistic, with values approaching +1 or −1 indicating stronger separation between the two ROI groups.

For the comparison of Ki67-positive proportions between EGFR-positive and EGFR-negative tumor cells, the two cell subsets were measured within the same five ROIs and compared using a two-sided paired t-test. The effect size was quantified using paired Cohen's d, calculated as the mean paired difference divided by the standard deviation of the paired differences. The mean paired percentage-point difference and d were reported as complementary

effect-size summaries. A two-sided P value <0.05 was considered statistically significant. All ROI-based analyses were interpreted as exploratory within-specimen comparisons; the ROIs represented spatial sampling units and not independent biological replicates.

Reference

[1] Xiao C, Zhou R, Chen Q, et al. Integrative spatial profiling pipeline for determining TME architectures in archival clinical specimens using CmTSA superplex technology. Cell Discov. 2026;12(1):16. doi:10.1038/s41421-026-00874-9.

[2] Uwe Schmidt, Martin Weigert, Coleman Broaddus, and Gene Myers. Cell Detection with Star-convex Polygons. International Conference on Medical Image Computing and Computer-Assisted Intervention (MICCAI), Granada, Spain, September 2018.
